# The metabolic parameters based on volume in PET/CT are associated with clinicopathological N stage of colorectal cancer and can predict prognosis

**DOI:** 10.1186/s13550-021-00831-5

**Published:** 2021-09-06

**Authors:** Hidenori Kido, Shunsuke Kato, Kimihiko Funahashi, Kazutoshi Shibuya, Yousuke Sasaki, Yoshihisa Urita, Masaaki Hori, Sunao Mizumura

**Affiliations:** 1 Department of Radiology, School of Medicine, Faculty of Medicine, Toho University, 5-21-16 Omorinishi, Ota-ku, Tokyo, 143-8540 Japan; 2 Department of Oncology, School of Medicine, Juntendo University School of Medicine, 2-1-1 Hongo, Bunkyo-ku, Tokyo, 113-8421 Japan; 3 Department of Surgery, School of Medicine, Faculty of Medicine, Toho University, 5-21-16 Omorinishi, Ota-ku, Tokyo, 143-8540 Japan; 4 Department of Pathology, School of Medicine, Faculty of Medicine, Toho University, 5-21-16 Omorinishi, Ota-ku, Tokyo, 143-8540 Japan; 5 Department of General Medicine and Emergency Care, School of Medicine, Faculty of Medicine, Toho University, 5-21-16 Omorinishi, Ota-ku, Tokyo, 143-8540 Japan

**Keywords:** Threshold, Colorectal neoplasms, ^18^F-fluorodeoxyglucose, Positron emission tomography/computed tomography, Prognosis, Biomarkers, Metabolic tumor volume, Total lesion glycolysis

## Abstract

**Background:**

A combination of positron emission tomography and computed tomography (PET/CT) is an important modality for the diagnosis of carcinoma. Metabolic tumor volume (MTV) and total lesion glycolysis (TLG) have been reported as metabolic parameters in PET/CT since the late 1990s, and they are expected to be useful in diagnosing diverse cancers and as prognostic biomarkers. We evaluated the potential of these parameters in the prognosis of colorectal cancer (CRC) by comparing them with conventional parameters, including the maximum standardized uptake value (SUVmax). We enrolled 84 patients who underwent surgery for CRC without distal metastasis between April 2015 and April 2019. SUVmax, MTV, and TLG were measured by ^18^F-fluorodeoxyglucose (FDG)-PET/CT. To find an optimal threshold value related to prognosis, the volume of interest in the primary carcinoma was measured at fixed relative and absolute thresholds based on SUVmax (30%, 40%, and 50%; 2.5, 3.0, and 3.5, respectively), tumor-to-liver standardized uptake ratios, TLR (1.0, 1.5, and 2.0), and SUV normalized to lean body mass, SUL (2.0, 2.5, and 3.0). After classifying the patients into two groups according to pathological N stage, the optimal threshold values of all metabolic parameters were compared between groups using a non-parametric comparison test.

**Result:**

The most suitable thresholds for MTV were a SUVmax of 3.5 and a TLR 2.0. TLG with a SUVmax value of 40% showed the most significant difference. The MTV standard uptake ratio of 2.0 was significantly associated with pathological N stage.

**Conclusion:**

Our results suggest that an MTV TLR 2.0 on PET/CT reflects pathological N stage in local patients with CRC.

## Background

^18^F-fluorodeoxyglucose positron emission tomography (^18^F-FDG PET) can be used to estimate the stage and recurrence of colorectal cancer (CRC) [1]. It has demonstrated promising accuracy in detecting colorectal masses and, when combined with computed tomography (^18^F-FDG PET /CT), superior sensitivity and specificity in detecting CRC [1–3]. ^18^F-FDG PET may thus facilitate prognosis for patients with CRC liver metastasis [4]. However, the accuracy of the diagnosis depends on the ability and experience of the medical practitioner, and its procedure might differ between hospitals. Denoting the maximal value in a voxel of a tumor as imaged with ^18^F-FDG PET, the SUVmax is a semiquantitative metabolic parameter that is often used to evaluate the prognosis of patients with CRC [2]. However, it does not adequately indicate the extent of glucose metabolism in volume-based glucose metabolic analysis. In contrast, MTV represents the tumor volume that exceeds SUV above a certain threshold, unlike CT volume, which provides only morphological measurement information, while TLG reflects the sum of FDG activity in a tumor above the same threshold. Since they are both based on volumes [5, 6], they may be superior to CT volume for accurate indication of tumor activity or the grade of malignancy and have greater prognostic value in determining tumor progression. However, the association of MTV and TLG with prognostic factors of CRC remains unknown. While the values of these parameters vary according to the threshold, an optimal threshold for measuring the volume of interest (VOI) is currently unavailable. From a clinical perspective, predicting N+ in patients with rectal cancer without metastasis indicates neoadjuvant chemotherapy as a treatment option according to NCCN guidelines, Version 1.2021. The guidelines for the treatment of CRC published by the Japanese Society for Cancer of the Colon and Rectum recommend the resection of main, intermediate, and pericolic lymph nodes to treat N+ disease and that of the intermediate and pericolic lymph nodes to treat T1N0 disease [7]. For the abovementioned reasons, it is important to use the determined presurgical risk of lymph node metastasis to inform the choice of neoadjuvant chemotherapy or range of lymphadenectomy. The overall accuracy of N-stage (lymph node metastasis) diagnosis by CT colonography using contrast medium ranges from 59 to 71% [8, 9]. In addition, the sensitivity and specificity of PET/CT were comprehensively estimated to be 42.9% and 87.9%, respectively, when preoperative lymph node involvement is detected in patients with CRC [10]. Therefore, a modality with higher accuracy is required. According to previous reports, MTV and TLG of the primary colon cancer were associated with T stage (depth of invasion) [11, 12], and were indicated by a Japanese study to be related with both T and N stages [13]. However, as the backgrounds of patients in these T- and N-stage groups were unknown, it cannot be concluded whether MTV and TLG were truly associated with these stages.

To validate the hypothesis that MTV and TLG are better biomarkers than existing parameters for predicting recurrence of CRC, we investigated the optimal threshold value of these parameters among the various thresholds reported in the literature to be useful in prognosis, as well as their association with CRC prognosis, after curative resection without preoperative adjuvant therapy. Further, we evaluated the association between lymph node metastasis (N stage) and SUVmax, MTV, and TLG in cases where there is no significant difference in the background (the diameter, histological type, and location of the primary tumor).

## Methods

### Data search and study selection

In this retrospective study, 110 consecutive patients who underwent surgery for CRC at Toho University Hospital between April 2015 and April 2019 after examination with FDG-PET/CT were enrolled. The exclusion criteria included the following: (1) underwent neoadjuvant chemotherapy or radiation therapy before the operation (n = 14) and/or did not have pathologically proven primary CRCs, (2) had distal metastasis (n = 3; M1), (3) had any other malignant tumor (n = 3), (4) were not evaluated for recurrence (n = 3), and (5) were recommended adjuvant chemotherapy (n = 3). Consequently, in the study, 84 patients were included.

The enrolled patients were classified into two groups based on the pathological N stage (Japanese Classification of Colorectal Carcinoma, 8th edition) [14]—N0 (no lymph node metastasis) and N1–N3. The histological types were classified into two groups—one group consisted of poorly differentiated carcinoma and mucinous adenocarcinoma and the other group consists of well-defined adenocarcinoma, adenocarcinoma with moderate differentiation, papillary adenocarcinoma, poorly differentiated carcinoma, and mucinous adenocarcinoma. The T stage was categorized into ≤ T2 or ≥ T3 based on criteria by the Japanese Association of Clinical Cancer Centers (5-year survival rate was > 95% in stages Tis, T1, and T2, and < 95% in T3 and T4 without metastasis) [15]. Accordingly, stage II or stage III were defined (5-year survival rate was > 95% in Stage 0, Stage I, and Stage II, and < 95% in Stage III and Stage IV).

### Acquisition of PET/CT image

Patients fasted for at least 5 h before undergoing the imaging test. For routine PET/CT (Biograph mCT flow20, Siemens Healthcare, Tokyo, Japan), 185 MBq FDG was administered. The CT machine was set at the following settings: voltage, 120 kV; rotation time, 0.5 s/rotation; pitch, 1.2; and automatic adjustment of electric current with automatic exposure control. PET data from the skull base to the ilium and the ilium to the knee were acquired at 1.1 cm/s and 1.3 cm/s, respectively, using continuous bed motion mode. We used the three-dimensional ordered subset expectation maximization algorithm (iterations, 2; subsets, 21) in combination with point spread function (PSF) and time of flight (CT attenuation correction; matrix size, 200 × 200; pixel size, 3.54; and Gaussian filter [full width at half-maximum = 6 mm]) for reconstruction of the PET image. The image was harmonized without PSF, and SUVs were measured. We used EQ PET software (Siemens Healthcare; filter = 5.8 m) to smoothen the digital input image data using a Gaussian filter. This process yielded an accurate SUV without degradation of image quality. Given that we analyzed the results with EQ PET software, which conformed to an EARL reconstruction, the outcomes should be comparable to those assessed with other scanners.

### Measurement of FDG metabolic parameters

A focus with ^18^F-FDG uptake greater than in the surrounding tissue, excluding any physiologically increased accumulations, was defined as a positive PET/CT finding. In axial, coronal, and sagittal sections, sufficiently wide volume boundaries were drawn to include the target lesion. Two nuclear medicine physicians independently defined the voxel of interest for target lesions and measured metabolic parameters on a three-dimensional image viewer (syngo.via, Siemens Healthcare). As the rate of concordance between the two reviewers was approximately 90%, we considered the results reproducible. In the case of multiple CRCs, we selected the VOI such that it included the region with the most advanced pathological T and N stages and measured its metabolic parameters. The margins of the target lesion inside the VOI were automatically delineated. Tumor segmentation was performed using relative thresholds of 30%, 40%, and 50% of the SUVmax (SUVmax of 2.5, 3.0, and 3.5, respectively). The threshold of 40% of the SUVmax of 2.5 was chosen because of its common use, while the others were selected based on their proximity to it. A VOI was placed in the liver to measure the background SUV. The colon close to lesions is engaged in physiological uptake that is expected to fluctuate more greatly than that of the liver. We suspected that the colon’s physiological uptake might affect metabolic parameters as a result. The criteria of PERCIST 1.0 concerning the use of FDG PET-CT for predicting treatment response recommended obtaining volume-based parameters with the use of liver SUV as a threshold to minimize the influence of inter-study variability of tumor SUV; the SUV mean plus 1 or 2 standard deviations (SD) of the background has been acknowledged as useful in determining parameter thresholds [16].

We placed a VOI approximately 3 cm (14 mL) in diameter on the normal right hepatic dome to determine the mean liver SUV (SUV_liver_). The tumor SUV normalized to the SUV of the reference liver (tumor-to-liver ratio [TLR]) was computed as the ratio of the maximum lesion SUV to SUV_liver_. Therefore, we used TLRs of 1, 1.5, and 2 as the background threshold. Lean body mass (LBM)-corrected SUV (SUL) was found to be a better quantitative method for obese patients than using body weight (BW) or body surface area. Therefore, we used SUL values of 2.0, 2.5, and 3.0 as thresholds. SUL was measured using James’ formula which relied on sex, height (cm), and total BW (kg) to estimate LBM—Men = 1.1 × BW − 128 × (BW/Height); Women = 1.07 × BW − 148 × (BW/Height). We defined MTV as the sum of voxels and calculated TLG as MTV multiplied by the SUV mean (the average SUV within a VOI). For metabolic evaluation, we also measured SUVmax, calculated using maximum activity values in a VOI placed manually over the visible area of the target lesion on each PET image. MTV and TLG values are significantly affected by the segmentation methods used. As the optimal prognostic thresholds of MTV and TLG in CRC have yet to be identified, we estimated them at our hospital and, based on our findings, determined whether other hospitals should evaluate their own standard optimal thresholds.

### Statistics

#### Identifying the optimal threshold for the association of MTV and TLG with recurrence within one year after surgery

Recurrence within the first year after surgery was assessed in patients with CRC by analyzing receiver operating characteristic (ROC) curves for MTV and TLG. PET parameters’ optimal cut-off values were defined to maximize sensitivity and specificity. In our hospital, clinical assessment and laboratory tests, including CEA・CA19-9, are performed every three months within the first two years following the surgical resolution of Stage II or III CRC, and CT scans of the chest, abdomen, and pelvis with IV contrast are conducted every three to six months. Alternatively, the abovementioned assessments and tests are performed every six months during the first two years following the surgical treatment of Stage I CRC, and the CT every six to twelve months within the same period. If the levels of CEA or CA19-9 exceeded the upper limits of their normal ranges (5.0 ng/ml, 37.0 U/ml) and were increasing, an additional CT was performed. According to the 2005 ASCO guidelines, patients with CRC who are at risk of recurrence after surgery and who could be candidates for curative-intent surgery, are recommended to undergo chest and abdomen CT. Additionally, a pelvic CT scan should be considered for rectal cancer surveillance, especially for patients who have not been treated with radiotherapy. Hence, these guidelines informed our selection of CT scans of the chest, abdomen, and pelvis with IV contrast. The most common site of local and distant recurrence is reportedly the liver [17], and many liver metastases in Japan reportedly occur within a year of surgery [18]. Early prediction of recurrence may make resection possible; hence, we limited our investigation of recurrence to the scope of a year. We confirmed the lack of the appearance of a tumor immediately after surgery and defined recurrence as the new appearance of a tumor, as confirmed on a follow-up CT scan performed according to schedule or after abnormal tumor markers (CEA or CA19-9) exceeded normal limits.

Based on the optimal cut-off value for MTV and TLG with the threshold value for each, obtained using ROC analysis, patients were separated into two groups (N0 and N+). We used the product-limit method and comparative test log-rank test to analyze the progression-free survival curves of these groups.

#### Association between PET/CT parameters and pathological N stage

We compared the increase in SUVmax, MTV, and TLG with pathological N stage (N0 or N1–3) with Wilcoxon signed-rank test. We used JMP 13 (SAS Institute, Inc., Cary, NC) to conduct all statistical analyses. For analysis, *p* < 0.05 was considered statistically significant.

If patients with CRC are suspected of having regional lymph node metastasis without distant metastasis before surgery or change in clinical stage from I or II to III, we considered them to be at high risk of recurrence. Determining the risk of recurrence helped to inform our decision to administer neoadjuvant chemotherapy or perform lymphadenectomy to avoid the recurrence.

## Results

### Study characteristics

**Fig. 1:**
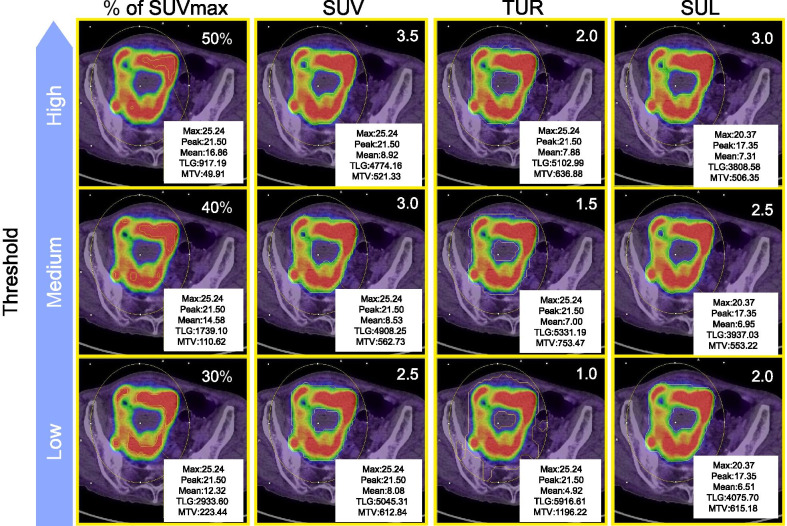
Thresholds in case of a higher SUVmax tumor. SUVmax, maximal standardized uptake value; SUV peak, peak standardized uptake value; SUV, mean standardized uptake value; MTV, metabolic tumor volume; TLG, total lesion glycolysis; TUR, tumor-to-liver uptake ratio; SUL, SUV normalized to lean body mass; VOI: volume of interest. VOIs in % of SUVmax were smaller than in other thresholds. For each threshold, the lower the value, the larger the VOI

**Fig. 2:**
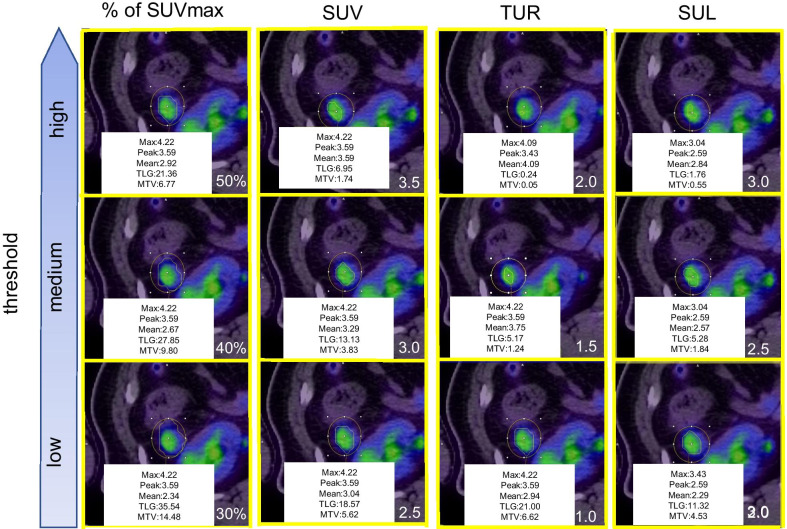
Thresholds in case of a lower SUVmax tumor. SUVmax: maximum standardized uptake value, SUV peak: peak standardized uptake value, SUV mean: mean standardized uptake value, MTV: metabolic tumor volume, TLG: total lesion glycolysis, TUR: tumor-to-liver uptake ratio, SUL: SUV normalized to lean body mass; VOI: volume of interest. VOIs for TUR2 were smaller than in other thresholds. VOIs in % of SUVmax were larger than in other thresholds. For each threshold, the lower the value, the larger the VOI

The study characteristics are shown in Table 1. The rate of poorly differentiated and mucinous types of cancer was 16.67% (14/84). We classified them into two groups according to the pathological N stage (N0 or N1–3). The groups differed significantly in pathological staging (*p* < 0.01); however, we detected no differences in the diameter, histological type, and location of the primary tumor. Figures 1 and 2 show the threshold in a relatively high SUVmax tumor and low SUVmax tumor, respectively.

**Table 1:** Patient characteristics

	N pos	N neg	*p*
Age (< 65 years/≥ 65 years)	4/12	24/44	0.560857
Sex (female/male)	10/6	24/44	0.053917
Primary lesion location			
Right colon/left colon	4/12	14/54	0.738752
Pathological staging (JCCC 8th edition)			
Tis + T1 + T2/T3 + T4	0/16	22/46	0.008809
Stage1 + 2/3 + 4	3/13	67/1	< 0.001
Histological types			
W/D + M/D + Pap / P/D + Muc	12/4	58/10	0.454159
Diameter			
≤ 4 cm/> 4 cm	4/12	34/34	0.095556

Pathological staging: T stage and Stage in Japanese Classification of Colorectal Carcinoma (8th edition), W/D: well-defined adenocarcinoma, M/D: adenocarcinoma with moderate differentiation, P/D: adenocarcinoma with poor differentiation, Pap: papillary adenocarcinoma, Muc: mucinous adenocarcinoma. *P*-values were calculated with Fisher’s exact test

All patients were classified into two groups according to pathological N stage, and data concerning age, sex, primary lesion location, pathological staging, histological types, and diameter were collected from each group; significant differences in pathological staging were found in both, but not in age, sex, primary lesion location, histological types (mucinous/poorly adenocarcinoma and others), or diameter. MTV was used as the average size, The SUV max of 2.5 for MTV was 49.99 (2.04–612.84). With the exception of one patient, all patients with CRC relapse had distant relapse.

### Outcomes

#### Identifying the optimal threshold for the association of MTV and TLG with recurrence within one year after surgery

For the ROC analysis of MTV and TLG in CRCs, the areas under the curve were 0.9506–0.9589 and 0.7880–0.8038, respectively; for the optimal prediction of recurrence, the cut-off values for the prognosis of recurrence were 16.52–75.94 and 234.93–560.11, respectively.

**Fig. 3:**
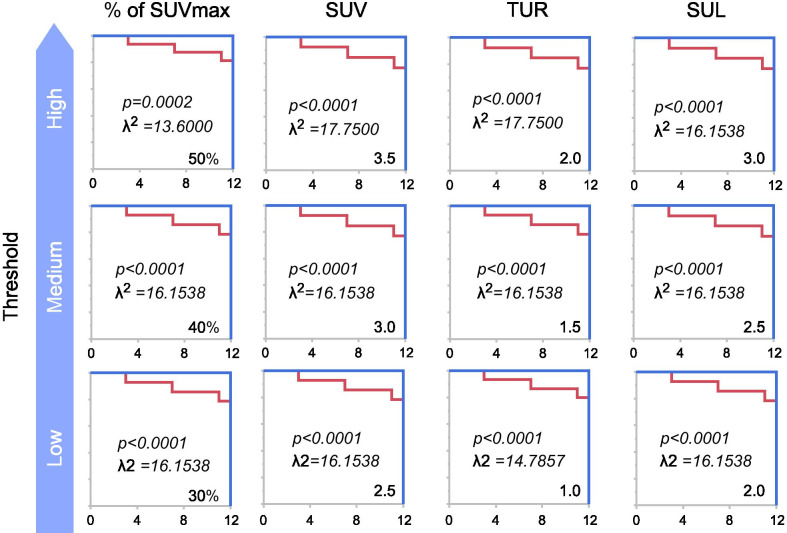
Relapse-free survival curve showing MTV for each threshold. SUVmax, maximal standardized uptake value; MTV, metabolic tumor volume; TLG, total lesion glycolysis; TUR, tumor-to-liver uptake ratio; SUL, SUV normalized to lean body mass; ROC: receiver operating characteristic curve. Relapse-free survival curves of the patients were divided by values below and above the cut-off value obtained using ROC analysis. For all the parameters for MTV, particularly the SUVmax of 3.5 (MTV 3.5, and TLR of 2 (MTV-TLR2), the values above the cut-off value were more frequently associated with recurrence than the values below the cut-off value

**Fig. 4:**
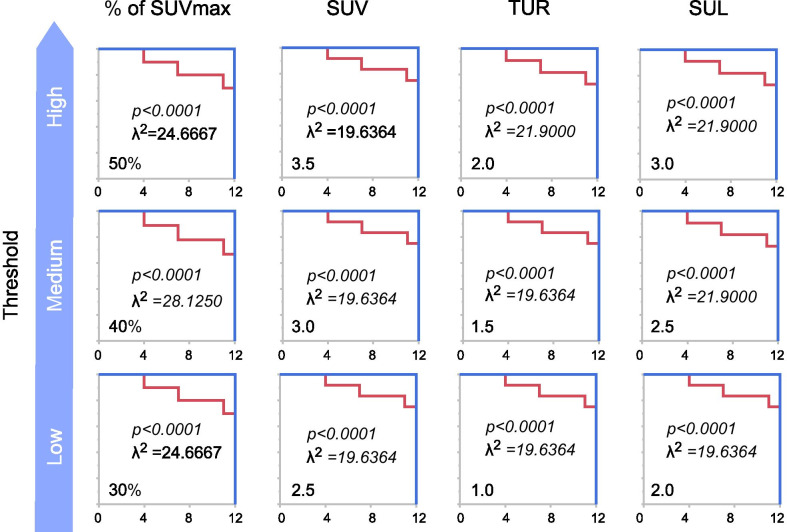
Relapse-free survival curve showing TLG for each threshold. SUVmax, maximum standardized uptake value; MTV, metabolic tumor volume; TLG, total lesion glycolysis; TUR, tumor-to-liver uptake ratio; SUL, SUV normalized to lean body mass. Relapse-free survival curves of the patients were divided by values below and above the cut-off value obtained using ROC analysis. For all the parameters for TLG, particularly 40% of tumor SUVmax for TLG (TLG 40%), the values above the cut-off value were more frequently associated with recurrence than the values below the cut-off value

When patients were divided according to optimal cut-off values, the relapse-free survival curves in the subgroups differed significantly between the values above and below the optimal values of PET parameters for each threshold, especially for 40% of tumor SUVmax for TLG (TLG 40%), SUVmax of 3.5 for MTV (MTV 3.5), and TLR of 2 for MTV (MTV-TLR2) (Figs. 3 and 4). There was no significant difference between the group with poorly differentiated and mucinous types and those without (*p* = 0.5169) in TLG 40%, but TLG 40% of their types tended to be bigger.

#### Relationship between PET/CT parameters and pathological N stage

**Fig. 5:**
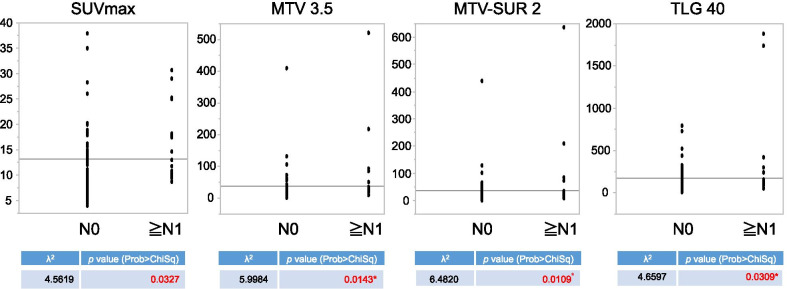
Relationship between metabolic parameters and lymph node metastasis. SUVmax: maximal standardized uptake value, MTV 3.5: SUVmax of 3.5 for metabolic tumor volume, MTV-TLR2: tumor-to-liver uptake ratio of 2 for metabolic tumor volume, TLG 40%: 40% of tumor SUVmax for total lesion glycolysis. The increase in glucose metabolic parameters with the progression of the N stage revealed that PET parameters (SUVmax, MTV, and TLG), especially TLR of 2 for MTV (MTV-TLR2), were significantly different between pathological N0 and other N stages

All parameters (SUVmax, MTV 3.5, MTV-TLR2 and TLG 40%) in the N-positive (N1-3) group were more significant than those in the N0 group, as revealed by the Wilcoxon signed-rank test (SUVmax χ^2^ = 4.5819, *p* = 0.0327 in SUVmax; MTV 3.5 χ^2^ = 5.984, *p* = 0.0143 in MTV 3.5; MTV-TLR2 χ^2^ = 6.4820, *p* = 0.0109 in MTV-TLR2;TLG 40% χ^2^ = 4.6597, *p* = 0.0309 in TLG 40%; Fig. 5).

## Discussion

In this study, the diameter, histological type, and location of the primary tumor were not significantly different between N-negative (N0) or N-positive (N1–3) groups. The metabolic parameters, especially MTV-TLR2, differed more in the N-positive group than in the N-negative group. Primary carcinoma is known to infiltrate intratumoral lymphatic vessels and spread through lymph nodes, and these metabolic parameters are expected to be related to intratumoral lymphatic vessel invasion. In non-small lung cancer (NSCLC), the high FDG uptake of primary carcinoma is related to invasion and lymph node metastasis [19], and both uptake and tumor size were significant factors [19]. Therefore, MTV or TLG, signifying the spread of high-grade tumors, was strongly related to lymph node metastasis [20, 21]. However, there are only a few reports on the correlation between metabolic parameters and intratumoral invasion of lymphatic vessels or lymph node metastasis in CRC [13]. Previously, 40% of tumor SUVmax for TLG (TLG 40%), but not MTV, was significantly related with pathological T stage in CRCs on the left side of the large intestine [22]. Therefore, TLG 40%, and not MTV 40%, was expected to be related to its invasion and node metastasis. Because of the different thresholds in MTV, MTV-TLR2 are expected to be more strongly related to T stage in CRC than other PET parameters. Further investigation is needed to explore whether MTV-TLR2 or TLG 40% are related to the depth of invasion and intratumoral vessel invasion in CRC. Based on our results, we expect MTV-TLR2 of the primary tumor to serve as a biomarker to predict its metastasis in patients with CRC. Further, we expect it to determine the suitability of neoadjuvant chemoradiation, neoadjuvant chemotherapy, or range of lymphadenectomy, even if the metastasis has low FDG uptake and poor morphological change. In CRC, the optimal threshold of metabolic parameters associated with prognosis was unknown, and MTV and TLG values change depending on the threshold. Therefore, we evaluated thresholds (SUVmax, MTV, and TLG) that were closely related to relapse—TLG 40% was the most common parameter related to recurrence within the first year after surgery, and MTV 3.5 and MTV-TLR2 performed better than MTV with other thresholds. A threshold of 36–44% was found to produce volumes similar to those measured from CT for lung cancer lesions > 4 mL [16]. Consequently, thresholds of 40–42% are most commonly used to measure MTV, and we confirmed that TLG 40% was associated with prognosis. However, the prediction of prognoses was often limited by common underestimations of the tumor volume with heterogeneous uptake (such as necrotic cores) on relative thresholds. In addition, Erdi et al. [23] reported that they overestimated small lesions with a low signal-to-background ratio using a fixed relative threshold. Figure 1 shows that VOIs in SUV %, in particular 50%, were smaller than other thresholds in a tumor with a high SUVmax; the inverse is shown in Fig. 2. The fixed relative method did not show the exact range of tumors with various sizes and signal-to-background ratios. However, the TLG threshold of 40% was observed to be the most prognostic parameter in this study. We therefore, speculated that TLG 40% could predict higher FDG uptake associated with poor prognosis in cases of high-SUVmax tumors, as well as tumors with low density and limited morphological changes with a tendency to be scattered throughout the body and are associated with poor prognosis in low-SUVmax tumors. In addition, their causes might predict distant relapse (e.g., in the lung, liver, peritoneum). Our findings indicated that MTV 3.5 and MTV TUR2 were more prognostic than were the other thresholds in MTV. Based on the results of earlier studies that used absolute cutoff values to distinguish benign from malignant lung nodules [24–26], we selected an SUVmax threshold value of 2.5. A meta-analysis found that parameters with an SUVmax threshold of 2.5 were significantly related to prognosis [27]. Various non-malignant lesions, such as inflammation and infection, may cause an increase in FDG uptake. In this study, MTV 3.5 was more prognostic than was MTV 2.5 in CRC; colon particular physiological uptake could be considered as a cause. Figure 1 shows that VOIs in SUV 3.5 were larger than the relative thresholds in a tumor with a high SUVmax; the inverse is shown in Fig. 2. If a tumor had an intense FDG uptake (such as SUV of > 15) according to an absolute method, their parameters could easily be overestimated by the spillover effect. Figure 1 also shows the overestimation of the absolute method. The degree of the overestimation was comparatively small and might not influence the prognosis. As a result, we considered MTV 3.5 to be more prognostic than MTV with the other thresholds, except for MTV TLR2. The most prognostic threshold in MTV was different in TLG. MTV was related more strongly related with regional lymph node metastasis than with TLG, and TLG was related more strongly related with relapse than MTV. These findings informed the conclusion that MTV was related more strongly with the depth of invasion or intratumoral invasion of lymphatic vessels than was TLG and that TLG was related more strongly with distant relapse than was MTV. These reasons might lead to the differences in the most prognostic threshold between MTV and TLG. Further investigation is needed to explore whether MTV or TLG is related to the depth of invasion, intratumoral vessel invasion, and distant relapse. To measure SUV, previous investigators placed a region of interest in the liver or mediastinal blood pool, and the mean SUV plus one or two standard deviations was then used as the background threshold [16]. Lesions with heterogeneous tracer uptake are often underestimated using the relative threshold; however, the liver can be subtracted as the background-based threshold, and its tumor voxels can be included in the MTV or TLG [28]. As seen in Fig. 1, a VOI of the liver background threshold method resulted in greater overestimation than did the relative method. However, a VOI of the liver background threshold method, in particular TUR2, showed greater underestimation than did that of the relative method (Fig. 2). The degree of the overestimation and underestimation were also comparatively small, and we therefore, considered MTV TUR2 to be more prognostic than the others in MTV, except for MTV 3.5. However, this method is more time consuming, and its reproducibility was lower than that at other thresholds. We also evaluated the SUL of the lesion at baseline. If FDG was distributed evenly inside the body, the SUV would be calculated as 1.00. However, since there are physiologically high and low metabolic organs, the volume of FDG distribution and SUV can be overestimated with increased body fat. Hence, the use of SUL has previously been advocated [28]. There were few obese patients with CRC, and SUL might be not more strongly associated with prognosis than are other thresholds in this study.

Our study had some limitations. We studied retrospectively in a single center and there could be a patient selection bias. Although we performed the clinical assessment and laboratory tests including CEA CA19-9 and CT scans based on our hospital’s protocol, MRI and chest X-rays might also be performed for follow-up after colorectal cancer surgery, and follow-up including those tests could detect more cases of recurrence. We defined prognosis based on recurrence within the first year after surgery in CRC. Recurrence might be observed one year after colorectal cancer surgery, numerous new medications have been developed, recurrence might be delayed, and a longer follow-up period might be required. The relationships between metabolic parameters using original thresholds were estimated. As the background threshold, mediastinal blood pool, or adipose tissue near the tumor might be used, or another numerical value such as 2.0 might be used as the fixed relative threshold, so research including those thresholds is needed. Depending on the various sizes and signal-to-backgrounds, results could be greatly misleading by when using a fixed relative threshold. In such cases, it cannot be ruled out that other thresholds, such as the background threshold, might be more prognostic than a fixed relative threshold. In the future, the connection between the parameters and overall survival should be examined over a longer period with a multicenter common protocol, including MRI and chest X-ray in addition to blood and CT tests, as new medications have decreased mortality rates. The relationship between the optimal threshold of glucose metabolic factors among more thresholds, associated with prognosis and conventional high-risk factors, including lymph node metastasis, should be examined in a prospective multicenter study with a larger patient population exhibiting high mortality and a longer follow up of these patients will provide more definitive insight into the realistic value of MTV and TLG. Subgroup analysis will be performed for each size and signal-to-background, and it will be necessary to evaluate how the analysis will change in relation to prognosis.

## Conclusion

In conclusion, MTV-TLR2 was more strongly related to pathological lymph node metastasis than the other conventional glucose metabolic parameters. It may serve as a prognostic factor for relapse after surgery and a useful biomarker for guiding an appropriate treatment for CRC.

## Data Availability

All data generated or analyzed during this study are included in this published article and its supplementary information files. Anonymized data will not be made available upon request.

## Abbreviations

PET: Positron emission tomography
^18^F-FDG: ^18^F-fluorodeoxyglucose
CRC: Colorectal cancer
SUVmax: Maximum standardized uptake value
MTV: Metabolic tumor volume
TLG: Total lesion glycolysis
VOI: Volume of interest
OLM: Occult lymph node metastasis
PSF: Point spread function
SUV_liver_: Mean liver SUV
TLR: Tumor-to-liver ratio
LBM: Lean body mass
SUL: LBM-corrected SUV
ROC: Receiver operating characteristic curve
OLM: Occult lymph node metastasis
SUR: Standard uptake ratio
TLG 40%: 40% Of tumor SUV max for TLG
MTV 3.5: SUV max of 3.5 for MTV
MTV-TLR2: TLR of 2 for MTV
NSCLC: Non-small lung cancer
GLUT1: Glucose transporter 1
EGFR: Epidermal growth factor receptor
PTEN: Phosphatase and tensin homolog
